# Correction: Structural, electrical, and optical properties of NiO films for surface-enhanced Raman spectroscopy applications

**DOI:** 10.1039/d5ra90083b

**Published:** 2025-06-30

**Authors:** Long Nguyen Hoang, Le Huu Bao, Tran Le

**Affiliations:** a Faculty of Physics & Engineering Physics, University of Science, VNU-HCM Ho Chi Minh City Vietnam ltran@hcmus.edu.vn; b Vietnam National University Ho Chi Minh City Ho Chi Minh City Vietnam

## Abstract

Correction for ‘Structural, electrical, and optical properties of NiO films for surface-enhanced Raman spectroscopy applications’ by Long Nguyen Hoang *et al.*, *RSC Adv.*, 2025, **15**, 17365–17376, https://doi.org/10.1039/D5RA02866C.

The authors regret that the affiliations were incorrectly shown in the original manuscript. The corrected list of affiliations is as shown herein.

The Royal Society of Chemistry apologises for these errors and any consequent inconvenience to authors and readers.

